# Is there any relationship between the menopause transition and dizziness?

**DOI:** 10.1016/j.bjorl.2026.101763

**Published:** 2026-02-05

**Authors:** Lucas Resende Lucinda Mangia, Roseli Saraiva Moreira Bittar

**Affiliations:** a Universidade Federal do Paraná, Department of Ophthalmology and Otorhinolaryngology, Curitiba, PR, Brazil; b Universidade de São Paulo, São Paulo, SP, Brazil

**Keywords:** Climacteric, Dizziness, Vestibular diseases, Labyrinth diseases, Menopause

## Abstract

**Objectives:**

This review aims to study the impact of menopause transition on the vestibular system.

**Methods:**

A broad systematic search of biomedical databases was performed. Papers investigating the effects of changes in female sex hormones on the vestibular physiology or the relationship between these changes in midlife women and vestibular manifestations were reviewed. Data from basic science, epidemiological studies and clinical investigations were organized and elaborated. The authors also addressed the gaps and limitations of current literature.

**Results:**

Hormonal modifications during menopause transition seem to affect the vestibular system. Accordingly, epidemiological investigations describe a remarkable prevalence of vestibular disorders in climacteric women. However, clinical studies exploring this relationship are scarce, and little is known about the clinical and pathophysiological aspects of these diseases. The predisposition to vestibular disturbances during the menopause transition might be the result of an intricate combination of hormonal effects in the nervous system. These effects encompass changes in energy metabolism, immune and inflammatory regulation, modulation of several neurotransmitter systems, integrity of microcirculation and neuroprotection and neuroplasticity pathways. As a result, structural consequences within the inner ear, changes in the transduction and modulation of peripheral signals and dysregulation of central vestibular circuits may occur. Additionally, patients may present with signs of disturbances in sensory processing or difficulties in recovering from functional damages to the vestibular system. These events may contribute to the manifestation of individual vulnerabilities, causing sensory complaints, and the onset or worsening of vestibular problems. Preliminary data also corroborate the impact of menopause transition in specific vestibular diseases.

**Conclusion:**

There is a theoretical basis for understanding the menopause transition as a facilitating condition for vestibular symptoms and diseases. However, clinical studies addressing these issues are scarce. Exploring them is a promising field of investigation that could lead to improvement in care for vestibular patients.

## Highlights

• The literature shows the impact of female sex hormones on the nervous system.
• Estradiol acts to modulate physiological functions related to vestibular activity.
• There is a high prevalence of vestibular symptoms and diseases in midlife women.
• Hormonal changes in menopause transition influence specific vestibular disorders.
• Understanding how menopause affect vestibular patients may help improve their care.

## Introduction

The effects of female sex hormones on the vestibular system are suggested by several studies. During the Menopause Transition (MT), in particular, clinical experience and epidemiological data point to a high prevalence of vestibular problems. Up to 50 %–60 % of climacteric women reported dizziness in broad population surveys.^1^^,^^2^ A Brazilian study also highlights the significant impact of vestibular complaints on the quality of life in these women.^3^ Despite the overall progress in basic research, little is known about the clinical manifestations related to the vestibular system during the menopausal transition.

The universal criteria established for vestibular disorders are useful for standardizing clinical investigations. However, they provide a narrow view of vestibular patients and should not prevent the study of shared mechanisms underlying different conditions. The Bárány Society itself recognizes the importance of considering these factors, which might include hormonal changes, in both vestibular research and clinical reasoning.^4^

## Methods

This narrative review aimed to investigate the influence of hormonal alterations during MT on vestibular symptoms and disorders. To do so, a thorough and systematic search of biomedical databases, including PubMed, Embase, Web of Science and MEDLINE was performed. A structured search method was employed and included combinations of the following keywords: “menopause transition”, “hormonal changes”, “estradiol”, “progesterone”, “dizziness”, “vertigo”, “vestibular system” and “vestibular disorders”.

First, the authors reviewed both the titles and abstracts of all the retrieved articles. Only papers published in English, Spanish or Portuguese were considered. Secondly, they included those investigating the effects of modifications in estrogens and/or progesterone on the vestibular system. Also, studies on the relationship between hormonal changes in climacteric women and vestibular manifestations were considered. Eligible articles were read in full text and cross-checked by the authors. Findings were elaborated and presented comprehensively, but with a rational approach. Hence, data from basic science, epidemiological studies and clinical investigations were organized and summarized in a structured format to enable a full view on the topic. Of note, most investigations with human subjects included in this review were individual case-control studies (evidence level 3b) of limited methodological quality.

## Results and discussion

### The menopause transition

Natural menopause is the permanent cessation of menstruation after 12 consecutive months of amenorrhea without any presumable cause.^5^ It is a specific event within the MT. This period is overall recognized by irregular menstrual cycles, hormonal changes, and clinical manifestations known as the climacteric syndrome.

There are two main trends in the hormonal environment during the transition: greater unpredictability in the variations of Estradiol (E2) and progesterone during each menstrual cycle, and progressive reduction in their average levels, with a secondary increase in serum Luteinizing Hormone (LH) and Follicle-Stimulating Hormone (FSH).

The climacteric syndrome encompasses a range of complaints including sleep disturbances, vasomotor symptoms, mood changes, and cognitive difficulties. These symptoms suggest that the MT is a period of neurological adjustments, with repercussions of hormonal fluctuations on the central and peripheral nervous systems. Even small disturbances during this process can induce the expression of innate vulnerabilities, with functional consequences.^6^^,^^7^

### Epidemiological evidence and limitations

Initial clinical investigations indicate that the MT might be associated with disturbances in balance and spatial perception. Thus, dizziness is frequently reported by midlife women in population-based samples.^1^^,^^8^^,^^9^ Besides, a significant correlation between vestibular manifestations and climacteric vasomotor symptoms has been reported.^10^^,^^11^

However, significant biases limit definitive conclusions from these preliminary data. There are no detailed studies on the features of vestibular symptoms during the MT. Yet, many health domains that also affect the vestibular and perceptual pathways, such as sleep, cognition, and mood, are usually impaired during this period. It has not been determined to which extent the impact of menopause on vestibular disorders could be actually secondary to these impairments.

### Basic science and limitations

Estrogens modulate multiple cell functions. To do so, they use an array of receptors and signaling pathways, which generate molecular and genomic responses. There are three types of estrogens: Estrone (E1), 17β-Estradiol (E2) and Estriol (E3). During reproductive years, E2 is the most abundant and potent circulating estrogen. Basic science data suggest that the fluctuation and privation of E2 affect the vestibular system. However, considering only the effects of E2 might underestimate the actions of E1 and E3. Yet, their physiological roles, as well as those of progesterone, FSH and LH is far less documented. Finally, the net effect of sex hormones is a matter of greater speculation.

During menopause, apart from progressive ovarian failure, many other physiological changes occur. They make it difficult to draw sharp conclusions in the field. Moreover, basic science studies frequently use ovariectomy in rodents as a model of menopause. However, natural menopause is a more complex process, so that the transposition of findings from animal research should be interpreted with due caution.^12^

In brief, it is possible to make convincing hypotheses about the impact of MT on the vestibular system. These hypotheses consider the effects of E2 in the production and processing of vestibular signals. Nevertheless, this is an evolving field of research, where scientific facts tend to be transitory.

### Possible mechanisms underlying female sex hormones effects on the vestibular system

#### Effects on energy metabolism

**Fig. 1:**
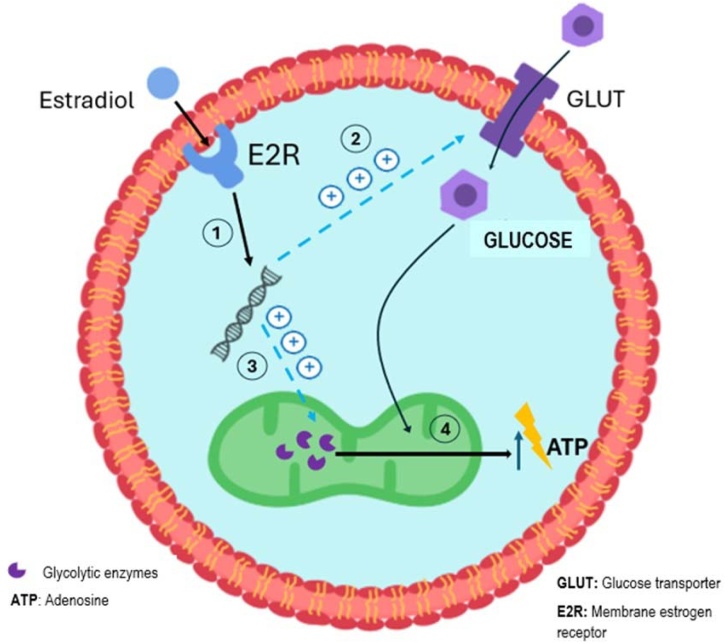
Schematic view on the role of estradiol on the energy metabolism within cells in the nervous system. It includes genomic actions (1) to favor the glucose uptake by membrane transporters (2) and stimulation of both the expression and activity of different glycolytic enzymes (3). These effects converge to increase energy production within the mitochondria (4).

Estrogen receptors are widely distributed in the Central Nervous System (CNS). More recently, estrogens have been implicated in controlling CNS energy metabolism.^13^^,^^14^ Neurons and astrocytes use glucose as the primary energy source, and estrogens promote the use of carbohydrates as substrates. Female aging leads to changes that facilitate a transition from an efficient glucose-based phenotype to a less adequate one, more dependent on ketones and fatty acids.^15^ Accordingly, during MT, studies document a decline in glucose metabolism in some cortical areas.^16^ The facilitatory effects of E2 on the bioenergetics of the brain are multiple, ranging from glucose transport to glycolysis (Fig. 1). Estradiol uses GLUT receptors to facilitate glucose absorption in the intestine and its extraction from the blood to the brain.^17^ In neurons, estrogens directly modulate glucose metabolism. For example, E2 increases the activity of glycolytic enzymes and protects components of the mitochondrial bioenergetic network from oxidative stress.^18^^,^^19^

Systemic E2 safeguards pancreatic β-cells, reduces hepatic insulin clearance, increases peripheral insulin sensitivity, and prevents insulin resistance.^20^, ^21^, ^22^ All these effects further contribute to the availability and metabolism of glucose in the brain.

The inner ear is particularly vulnerable to these phenomena, given the high energetic demand to maintain the endolymphatic potential. Similarly, the hypometabolic state of the CNS may hinder the cortical ability to execute complex tasks, such as those related with vestibular processing and sensory integration.

#### Effects on the inflammatory balance

Estrogens act as potent anti-inflammatory agents.^23^^,^^24^ During MT, the decline in their levels induces a systemic inflammatory state.

Treatment with E2 inhibits the activation of the microglia in response to inflammation-inducing agents.^25^ The inflammasome is a multiprotein complex engaged in the activation pro-inflammatory cytokines. It is sensitive to estradiol, and was detected in the cerebrospinal fluid of postmenopausal women.^26^^,^^27^ The transcription factor NF-κB, which modulated the expression of several pro-inflammatory genes, is inhibited by E2.^28^ Studies show an increase in pro-inflammatory interleukins after menopause.^29^^,^^30^ Furthermore, postmenopausal women tend to have elevated white blood cell counts, with increased total lymphocytes and monocytes, and a qualitative alteration in the T-cell response with a reduction in the CD4-positive lineage.^31^

Persistent inflammation associated with the estrogen decline might be key to the onset or worsening of many neurological dysfunctions.^32^ The pathophysiology of many vestibular disorders includes inflammatory cascades. This is the case with Vestibular Migraine (VM) and Meniere’s Disease (MD), whose inflammatory backgrounds were recently described.^33^

#### Effects on neurotransmitter systems

**Fig. 2:**
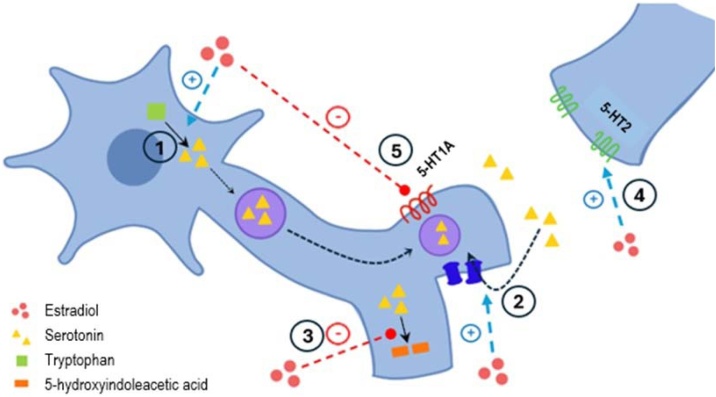
Molecular mechanisms of regulation of serotoninergic neurotransmission by estradiol in the central nervous system. Among them, one should highlight the stimulation of the synthesis (1) and reuptake (2) of serotonin; inhibition of the degradation of this neurotransmitter (3); increase in the expression and activity of post-synaptic receptors of subtype HT2 (4); and repression of expression and function of presynaptic receptors 5-HT1A (5).

Studies show that sex hormones, particularly estrogens, affect several neurotransmitter systems in the brain. In summary, E2 facilitates glutamatergic and serotonergic systems. It has varied effects on the opioid, dopaminergic, GABAergic, and noradrenergic pathways. On the other hand, progesterone primarily activates the GABAergic system and seems to modulate the actions of estrogens. These effects are complex and depend on many factors. They include actions on the synthesis, release, reuptake and clearance of each neurotransmitter, but also regulation of the production and activity of their receptors.^34^^,^^35^ For example, estrogens significantly alter the serotonergic tone through many mechanisms. Estrogens help produce 5-HT by stimulating the tryptophan hydroxylase. They also reduce 5-HT degradation by decreasing the activity of the monoamine oxidase.^36^^,^^37^ Additionally, estrogens inhibit serotonin reuptake, increasing 5-HT availability within the synaptic cleft.^37^^,^^38^ They also affect the gene expression of pre- and postsynaptic serotonin receptors, with cumulative effects on the serotonergic system.^37^ Fig. 2 schematically summarizes the key mechanisms by which estradiol may influence serotonergic tone.

Alterations in these neurotransmitter systems also presumably affect the vestibular pathways. Thus, diseases related to the efficacy of peripheral signal production and neural transmission may be facilitated. Similarly, these changes could impact recovery following vestibular injury or progression to chronic functional conditions.

#### Effects on neuroprotection and neuroplasticity

The neuroprotective effect of E2 has been extensively demonstrated. The mechanisms of E2-mediated neuroprotection are not fully understood. However, it seems to occur through the activation of signaling cascades that promote cell survival and induce the expression of anti-apoptotic genes.^39^

Animal studies confirm that E2 influences synaptic plasticity in many brain regions.^40^ Brain-Derived Neurotrophic Factor (BDNF) appears to be an important mediator of this action. BDNF is key for neuronal survival and differentiation. It exhibits neuroprotective effects in adverse conditions and modulates synaptic transmission and neuronal plasticity in the adult brain.^41^^,^^42^

There is a close relationship between E2 and BDNF. Studies show that the BDNF gene is estrogen-sensitive and that ovariectomy reduces its levels.^43^ In some areas, estrogen and BDNF likely work together to promote the formation of dendritic spines. The mechanism behind this action includes the modulation of gene expression via CREB (c-AMP response element-binding protein), a transcription factor that regulates several cellular responses. Electrophysiological studies conducted over more than three decades underline the effects of E2 and BDNF on synaptic plasticity.^39^

Many dynamic processes are essential to the optimal function of the vestibular system. They encompass visuovestibular calibration, vestibular compensation, and fine adjustments of responses to intense or repetitive stimuli.^44^ Synaptic plasticity has been demonstrated in vestibular nuclei, where it is likely involved in adaptive responses to these complex demands. Studies show that E2 affects synaptic transmission and neuronal excitability in the medial vestibular nucleus.^45^ It ultimately enhances the signal-to-noise ratio of the primary afferent neurons' responses compared to the resting activity of neighbor cells. Consequently, it might improve neuronal activation and reorganization in certain vestibular circuits.^44^^,^^45^

The cerebellum modulates vestibular function and contributes to accurate gaze stabilization and ocular responses to movements. Within the adult cerebellum, estradiol favors potentiation of glutamatergic neurotransmission between parallel fibers and Purkinje cells. This action was confirmed in studies of vestibulo-cerebellar pathways, where E2 improves the Vestibulo-Ocular Reflex (VOR) adaptation.^40^^,^^44^ Thus, E2 seems to modulate the VOR at different levels. It up- or downregulates the reflex gain by acting on the vestibular pathways or the cerebellum, respectively.^44^^,^^46^ Although the effects of the administration of E2 on synaptic plasticity at the Purkinje cell and the VOR adaptation were demonstrated in animal studies with ovariectomized mice and ERβ knock-out female mice, there still lacks parallel evidence from clinical investigations.^40^

Hormonal changes during MT could negatively affect neuroprotection and neuroplasticity in vestibular networks. They may compromise compensation and reduce the overall accuracy of vestibular responses, predisposing women to both functional problems related to suboptimal VOR adjustment, and maladaptive outcomes following injuries.

#### Microvascular effects

Pre-menopausal women are relatively protected against cardiovascular diseases. Atherosclerosis, a pivotal phenomenon when discussing this effect, involves complex mechanisms in which estrogens participate. E2 improves the circulating lipoprotein profile, modulates inflammation and has a positive impact on the vascular wall.^47^, ^48^, ^49^, ^50^

Oxidative stress results in endothelial dysfunction, vasculitis, and the accumulation of low-density lipoproteins in the vessel wall. Estrogens reduce the vascular impact of reactive oxygen species, by improving mitochondrial function. The cerebral microvasculature, including the blood-labyrinth barrier, is a critical target, as cerebral endothelial cells have particularly higher energy demands.^51^

Estrogen also modulate vascular reactivity. In cerebral circulation, chronic estrogen exposure reduces the vascular tone in an endothelium-dependent manner.^48^^,^^52^^,^^53^ This reduction is the result of the overall effect on the availability of vasorelaxant factors such as nitric oxide and prostacyclin, as well as of vasoconstrictors such as endothelin-1 and thromboxane A2.^54^ Moreover, estrogen withdrawal increases peripheral sympathetic neuronal activity and circulating norepinephrine levels, tipping the balance towards vasoconstriction after estrogen deprivation.^48^

**Fig. 3:**
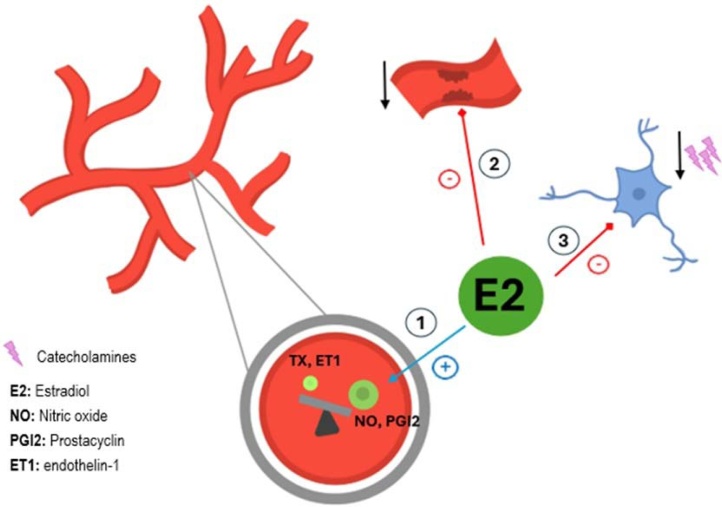
Some of the effects of estradiol in the vascular system. They include modulation of vasoactive molecules, favoring those with myorelaxant properties (1), protection against atherosclerosis (2), and reduction of the sympathetic tone (3).

The vascular effects of estradiol are summarized in Fig. 3.

In high-demand end-organs, E2 appears to prevent events related to endothelial dysfunction, vascular inflammation, endothelial repair, and angiogenesis. Consequently, E2 deprivation might predispose individuals to certain labyrinthine diseases, such as Benign Paroxysmal Positional Vertigo (BPPV) and MD.

### Female sex hormones and their impact on vestibular disorders: significance and limitations

Epidemiological studies consistently show that women are more affected by vestibular disorders. The largest study on this subject, including over 70 million people, revealed that women are more likely to report dizziness and vertigo.^55^ Epidemiological data also indicate a higher prevalence of certain specific vestibular disorders in women, such as BPPV, VM, postural perceptual persistent dizziness and motion sickness.^56^, ^57^, ^58^

The prevalence of MD peaks between the ages of 40 and 60, coinciding with the typical MT period in women. E2 can facilitate the loss of intravascular fluid into the extravascular space, predisposing to peripheral fluid retention. The specific localization of estrogen receptors in the spiral ganglion cells and in the stria vascularis, structures involved in the ionic and fluid balance of endolymph, supports this hypothesis.^59^ Jian et al. found lower estradiol levels in postmenopausal women with MD compared to controls. They also observed a correlation between hormone levels and the labyrinthine asymmetry in the caloric test.^60^ Postmenopausal women with MD who received add-on hormone therapy with estrogen and drospirenone showed better results in longitudinal balance assessment compared to their counterparts treated with vestibular rehabilitation alone. This additional benefit was attributed to the capacity of drospirenone, a progestogen with anti-mineralocorticoid activity, of reducing the fluid overload observed during MT.^61^

The relationship between migraine and sex hormones is evident, but not fully characterized. In general, migraines have higher incidence, frequency, duration, and degree of disability in women.^62^ The pathophysiology of migraine attacks is associated with trigeminovascular activation, sensitization of cortical sensory networks, changes in intracranial vasculature, and neuroinflammation.^63^

Vestibular migraine is a common condition that might account for a fair share of vestibular manifestations during MT. Hormonal variations could affect vestibular function in susceptible patients. In a controlled study, postmenopausal women with VM presented significantly reduced levels of estradiol, progesterone, and testosterone. Those with low estradiol levels reported higher handicap, more episodes, earlier symptom onset, and shorter disease-free survival.^64^

The effects of ovarian neurosteroids on neurotransmitter systems involved in the pathogenesis of VM is noteworthy.^62^ The hormonal impact on the serotonergic circuits could be particularly crucial due to the abundance of 5-HT across the trigeminovascular system.^65^ Ovarian steroids regulate neurotransmission in brainstem locations likely related to VM, such as the periaqueductal gray matter, locus ceruleus, and dorsal raphe nuclei.^66^ The role of estrogens in VM may be also secondary to their influence on cortical sensory processing networks. The fact that cortical excitability varies throughout the human menstrual cycle is consistent with this assumption.^66^

Calcitonin Gene-Related Peptide (CGRP) and substance P are neuropeptides involved in the molecular cascade associated with migraine. CGRP induces plasma extravasation, vasodilation, and mast cell degranulation and substance P is e engaged in neurogenic inflammation. The serum levels of both are sensitive to fluctuations in ovarian hormones.^67^^,^^68^

A promising site of pathophysiological abnormalities in VM and likely vulnerable to estrogen influence is the Efferent Vestibular System (EVS). This neural circuit is extensively described in animals but has still not gained enough attention in humans. It includes a neural network whose cell bodies are in the brainstem and project to the peripheral vestibular organ.^69^^,^^70^ In the labyrinth, the EVS synapses with primary vestibular receptors and vestibular afferent fibers. It seems to modulate the activity of the labyrinthine sensory organs and the firing rate of the afferent fibers.^69^ Thus, the EVS might be relevant for explaining how central mechanisms of migraine could eventually affect the periphery. Consistently, CGRP has been identified among the neurotransmitters released in the distal region of the EVS.^69^^,^^71^

Finally, fluctuations in estrogen levels may trigger episodes of VM also due to their effects on the microvasculature and in the sympathetic nervous system.^48^^,^^67^

**Fig. 4:**
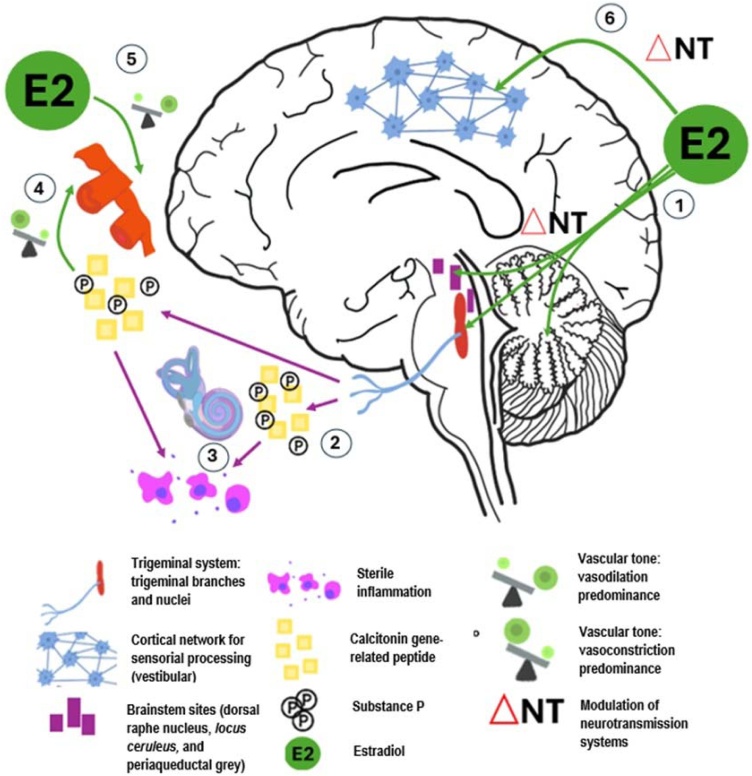
Possible actions of estradiol on the pathophysiological events of vestibular migraine during menopause transition. First, estradiol modulates neurotransmitter systems in involved sites, such as the cerebellum, the raphe dorsal nuclei, the locus ceruleus and the periaqueductal grey matter (1). Changes in estradiol levels may have an impact on the trigeminovascular activation, leading to the release of vasoactive neuropeptides, such as CGRP and substance P (2), and the induction of a distal neuroinflammatory response (3). This trigeminovascular activation predispose to vasoconstriction (4), which is directly counterbalanced by vasodilation effects of estradiol (5). Finally, estradiol might act on the cortical networks implicated in vestibular sensorial processing, where it could interfere with neuronal functioning and excitability (6).

Fig. 4 summarizes the potential effects of estradiol on the pathophysiological pathways involved in vestibular migraine.

The degeneration of otoconia may facilitate their detachment from the utricular macula and displacement to the semicircular canals. Women in menopausal transition are particularly prone to BPPV. One could argue that it could be due to concurrent hormonal changes on the integrity and physical properties of otoconia. Estrogen deficiency has been linked to structural damage and inadequate anchoring of these structures in the vestibule.^72^ Some authors believe that local disturbances in microcirculation are behind these findings.^73^

## Conclusion

In recent decades, an increasing body of evidence demonstrates the significant impact of female sex hormones, particularly estradiol, on the peripheral and central vestibular systems. Basic science studies suggest that estradiol plays a crucial role in the regulation of major physiological functions related to vestibular activity.

Alterations in these functions, may contribute to a range of neurological manifestations observed in individuals during certain life stages. The MT is particularly susceptible to cyclical and long-term hormonal variations. Accordingly, vestibular problems are consistently reported among midlife women.

Despite the growing evidence, vestibular symptoms in women during MT continue to be neglected by both the scientific community and health professionals. Future longitudinal studies should explore how hormonal transition affect vestibular patients and their clinical outcomes. Although underexplored, tackling hormonal female changes might be a potential avenue of management of vestibular disorders. Hence, trials exploring the benefits of treating menopausal syndrome in women with vestibular problems could further improve current understandings in this topic.

## ORCID ID

Lucas Resende Lucinda Mangia: 0000-0003-3443-3640

Roseli Saraiva Moreira Bittar: 0000-0001-8731-8908

Received 2 October 2024; accepted 27 October 2025

## Funding

This research did not receive any specific grant from funding agencies in the public, commercial, or not-for-profit sectors.

## Conflicts of interest

The authors declare no conflicts of interest.

## Data availability statement

The authors declare that all data are available in repository.
